# Are Physicians to Be Driven Back to Their Old Plan of Furnishing Their Own Medicines

**Published:** 1878-09

**Authors:** 


### Are Physicians to be driven back to their old plan of furnishing their own Medicines.

Our attention is often called to the behavior of our druggists in the putting up the prescriptions of physicians, but we have rarely thought worth while to mention things we could complain of, to our readers. We have always refused to write upon prescription papers having the card of a druggist upon it, thus treating all druggists alike, expecting honest fair dealing from all, not upon the ground of their honesty, but solely upon the conviction that they would see fairness and honesty *their* best policy. We thought to do a very fair thing by them in writing upon blank forms and allowing families to choose their own druggists, but we are now convinced of our mistake, and see how it is the *duty* of the physician to choose the druggist for his patient. It is neither safe or quite honest to allow patients in many cases to go to the druggist nearest or most convenient.

Hereafter we propose to tell patients where *not* to go for medicines, if we do not feel it necessary to direct where their prescriptions will be filled satisfactorily, and we propose to give our readers the reason why we do this for the benefit of both physicians and druggists in Buffalo and vicinity.

Recently a patient of ours called, bringing with him a bottle of medicine to find out if we thought it would be good for him. He said, I have always bought the pills you ordered at ——— drug store, but the last time I called Mr. Druggist said, "you have taken these pills a good while, they will get your system full of mercury, and I will give you something that will answer better in place of them," soon giving a bottle of Syrup Sarsaparilla, with a little Iod Potass, as near as we could judge.

The patient had primary and some secondary syphilitic symptoms still present, and Pil Hydrarg, v grs. at night, had been prescribed, and the necessity of continuing for a long time, carefully enjoined as the only safe method to pursue. The young rascal druggist had taken the whole case into his own hands, and it might be wicked to repeat what I said of him, and unnecessary to tell how earnestly I advised the unsuspecting patient to never visit again his quite elegant apartment in the city, unless he could give him a public whipping. I would not believe so infamous a story if he had not in hands the corroborative proof; but his whole statement showed that it could not be other than true. We have not yet given our readers the name of this ignorant unscrupulous scamp, but *next time we will* give the name in full.

In connection with other not quite so infamous cases, it shows us how our first duty to our patients, after advising them what medicines to take, is to tell them where to get it, or where *not to get it*, if we have any fears of being accused of interest in the trade. We have most worthy and reliable druggists, but they can not but suffer by having gigantic vagabonds in the same business, and called also druggists, but as a class we have great respect for their faithfulness, fidelity and undoubted trustworthiness. Such cases as the above must be made very rare, or the prescription business will be "closed up."

